# Preserved vascular integrity and enhanced survival following neuropilin-1 inhibition in a mouse model of CD8 T cell-initiated CNS vascular permeability

**DOI:** 10.1186/1742-2094-9-218

**Published:** 2012-09-18

**Authors:** Georgette L Suidan, Jonathan W Dickerson, Holly L Johnson, Theresa W Chan, Kevin D Pavelko, Istvan Pirko, Kim B Seroogy, Aaron J Johnson

**Affiliations:** 1 Neuroscience Graduate Program, University of Cincinnati, Cincinnati, OH, 45267, USA; 2 Department of Neurology, University of Cincinnati College of Medicine, Cincinnati, OH, 45267, USA; 3 Department of Neuroscience, Mayo Clinic, Rochester, MN, 55905, USA; 4 Department of Neurology, Mayo Clinic, Rochester, MN, 55905, USA; 5 Department of Immunology, Mayo Clinic, Gugenheim Building 4-11C, 200 First St. SW, Rochester, MN, 55905, USA

**Keywords:** Vascular endothelial growth factor (VEGF), CD8 T cell, CNS vascular permeability, Blood–brain barrier (BBB), Neuropilin-1 (NRP-1), Fetal liver kinase 1 (flk-1), FMS-related tyrosine kinase-1 (flt-1)

## Abstract

**Background:**

Altered permeability of the blood–brain barrier (BBB) is a feature of numerous neurological conditions including multiple sclerosis, cerebral malaria, viral hemorrhagic fevers and acute hemorrhagic leukoencephalitis. Our laboratory has developed a murine model of CD8 T cell-initiated central nervous system (CNS) vascular permeability in which vascular endothelial growth factor (VEGF) signaling plays a prominent role in BBB disruption.

**Findings:**

In this study, we addressed the hypothesis that *in vivo* blockade of VEGF signal transduction through administration of peptide (ATWLPPR) to inhibit neuropilin-1 (NRP-1) would have a therapeutic effect following induction of CD8 T cell-initiated BBB disruption. We report that inhibition of NRP-1, a co-receptor that enhances VEGFR2 (flk-1) receptor activation, decreases vascular permeability, brain hemorrhage, and mortality in this model of CD8 T cell-initiated BBB disruption. We also examine the expression pattern of VEGFR2 (flk-1) and VEGFR1 (flt-1) mRNA expression during a time course of this condition. We find that viral infection of the brain leads to increased expression of flk-1 mRNA. In addition, flk-1 and flt-1 expression levels decrease in the striatum and hippocampus in later time points following induction of CD8 T cell-mediated BBB disruption.

**Conclusion:**

This study demonstrates that NRP-1 is a potential therapeutic target in neuro-inflammatory diseases involving BBB disruption and brain hemorrhage. Additionally, the reduction in VEGF receptors subsequent to BBB disruption could be involved in compensatory negative feedback as an attempt to reduce vascular permeability.

## Findings

Disruption of the blood–brain barrier (BBB) is a hallmark feature of numerous neurological disorders as diverse as multiple sclerosis, stroke, epilepsy, infection, cerebral malaria and acute hemorrhagic leukoencephalitis (AHLE) [1-4]. Immune cells have been linked to central nervous system (CNS) vascular permeability and the ensuing neuropathology in all of the aforementioned conditions. Therefore, defining mechanisms by which immune cells promote BBB disruption is of paramount importance for understanding numerous neurologic diseases and developing therapeutic strategies to treat or prevent them. One cytokine, vascular endothelial growth factor (VEGF), has been strongly implicated in vascular permeability. Nevertheless, a complete mechanism by which VEGF contributes to BBB dysregulation under neuro-inflammatory conditions has yet to be elucidated [5]. VEGF has been implicated in the vascular permeability condition associated with Dengue hemorrhagic fever (DHF) as well as cerebral malaria [6,7]. Signaling by VEGF occurs via activation of its high affinity receptors. Among these, VEGF receptor flk-1 is thought to play the most prominent role in angiogenesis and vascular permeability as it is highly expressed on cerebral endothelial cells. Upon binding by VEGF, flk-1 undergoes phosphorylation at several tyrosine residues achieving an activated state [8]. Neuropilin-1 (NRP-1), a VEGF co-receptor, is a non-tyrosine kinase transmembranous glycoprotein that enhances the interaction of VEGF with flk-1 and amplifies the angiogenic effects of this signal transduction [9-11].

To study the interaction between antigen-specific CD8 T cells and the neurovascular unit (NVU) under neuro-inflammatory conditions, our laboratory has developed an *in vivo* model using a variation of the Theiler’s murine encephalomyelitis virus (TMEV) infection commonly used to study multiple sclerosis [12-15]. Through the use of this model system, we recently reported that VEGF mRNA is expressed predominantly in neurons, as early as two hours post-induction of CD8 T cell-initiated permeability. Detectable signal transduction was observed with phosphorylation of VEGF receptor flk-1 significantly increasing shortly thereafter. In these studies, we determined that inhibition of neuropilin-1 prevented increased phosphorylation of flk-1, reduced CNS vascular permeability, and preserved microvessel protein levels of the BBB tight junction protein, occludin. These observations supported a hypothesis in which CD8 T cell-initiated BBB disruption was occurring through neuronal expression of VEGF, VEGF signal transduction, and ultimately ablation of BBB tight junctions in CNS microvessels [16]. In the current study, we assessed flk-1 and flt-1 mRNA expression in the brain during the course of CD8 T cell-initiated CNS vascular permeability. We also determined the extent by which neuropilin-1 receptor inhibition reduces vascular permeability and hemorrhage formation as measured by gadolinium-enhanced T1-weighted and T2*-weighted magnetic resonance imaging (MRI), respectively.

CNS vascular permeability was induced as described previously [12]. Briefly, C57BL/6 mice were infected intracranially with 2 × 10^6^ PFU Daniel’s strain of TMEV. Seven days post-TMEV infection, mice were injected intravenously with 0.1 mg VP2_121-130_ (FHAGSLLVFM) peptide (GenScript Corp. Piscataway, NJ, USA) to initiate CD8 T cell-initiated BBB disruption. We have previously published that virus infection alone is not sufficient to induce overt BBB disruption. Seven-day TMEV-infected mice have minimal CNS vascular permeability, normal BBB tight junctions, and lack microhemorrhages [14-16]. Mice were euthanized at various time points following this induction to analyze gene expression events, vascular permeability, hemorrhage and overall survival. All experiments were approved by the Institutional Animal Care and Use Committee of the University of Cincinnati.

**Figure 1:**
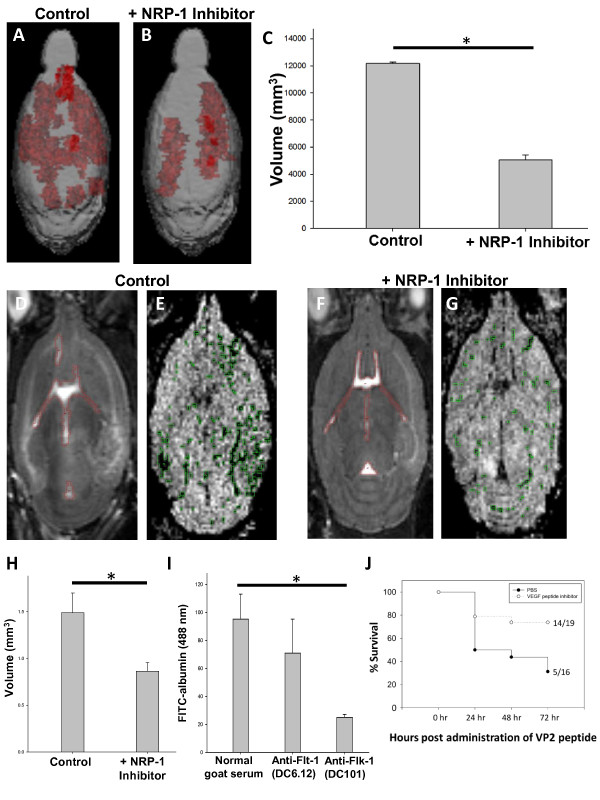
**NRP-1 inhibition reduces central nervous system (CNS) vascular permeability, microhemorrhage formation, and morbidity following induction of CD8 T cell-initiated blood–brain barrier (BBB) disruption.** We present the three-dimensional volume of gadolinium leakage as measured using T1-weighted magnetic resonance imaging (MRI) in a representative animal treated with (**A**) phosphate-buffered saline (PBS) or (**B**) NRP-1 inhibitor, 24 hours post-induction of vascular permeability with intravenous injection of VP2_121-130_ peptide. In (**C**), we demonstrate reduced three-dimensional volumes of gadolinium leakage in animals receiving NRP-1 inhibitor as compared to sham PBS-treated controls (*P*  < 0.001). T2 MRI was performed on animals receiving (**D**) sham PBS treatments or (**F**) NRP-1 inhibitor. Following these scans, the ventricle size was determined (red outline) and subsequently not included in the analysis of microhemorrhage area (green outlines) determined in (**E**) PBS-treated and (**G**) NRP-1 inhibitor-treated groups analyzed by T2*-weighted MRI. Using this method of analysis, we observed reduced microhemorrhage in NRP-1 inhibitor-treated animals as compared to PBS-treated controls (*P* = 0.023). All MRI scans were analyzed blind before breaking the code of each treatment group. (**I**) Quantification of FITC-albumin leakage into the brain reveals that pretreatment with DC101 antibody to flk-1, but not DC6.12 antibody to flt-1, is effective in reducing CNS vascular permeability (*P* = 0.003). In (**J**), administration of NRP-1 inhibitor enhances survival of mice undergoing CD8 T cell-initiated CNS vascular permeability (*P* = 0.014).

To determine the contribution of neuropilin-1 inhibition in reducing CNS vascular permeability, microhemorrhage, and overall survival, 3 mg of ATWLPPR peptide or PBS (sham treatment) was intravenously injected at −30 minutes, 3 hours, 6 hours and 9 hours post-administration of VP2_121-130_ to initiate CD8 T cell-initiated BBB disruption. Twenty-four hours post-VP2_121-130_ peptide administration, mice were scanned using gadolinium-enhanced T1-weighted MRI to assess CNS vascular permeability and T2/T2*-weighted MRI to assess hemorrhage formation according to our previously published methods [14]. Analyze 10 software developed by the Mayo Clinic was used to quantify the three-dimensional volume of gadolinium leakage from vasculature as well as the volume of microhemorrhage. Treatment with NRP-1 inhibitor (n = 4) markedly reduced three-dimensional gadolinium enhancement leakage when compared to PBS-treated controls (n = 2) (P  < 0.001, Student’s *t*-test) (Figure 1A-C). Treatment with NRP-1 inhibitor (n = 5) also significantly reduced microhemorrhage formation when compared to treatment with PBS (n = 4) (*P* = 0.023, Student’s *t*-test) (Figure 1D-H). In addition, inhibiting NRP-1 significantly increased survival of mice administered VP2_121-130_ peptide undergoing CD8 T cell-initiated BBB disruption (*P* = 0.014, Kaplan-Meier survival curve analysis). Mice were monitored for 72 hours post-administration of VP2_121-130_ peptide and did not receive additional NRP-1 inhibitor treatment past the 9-hour time point. We also determined that treatment with NRP-1 inhibitor did not alter viral loads when compared to treatment with PBS using quantitative real time (RT)-PCR to detect viral RNA as a ratio to actin mRNA (PBS group mean = 57027 ± SD 12287, 3 mg NRP-1 inhibitor mean = 43687 ± 15474, *P* = 0.519, Student’s *t*-test). Additionally, vascular permeability was analyzed by quantifying fluorescein isothiocyanate (FITC)-albumin leakage into the brain in mice administered normal goat serum (n = 2; 0.750 mg), DC101 antibody to flk-1 (n = 4; 0.500 mg), or DC6.12 antibody to flt-1 (n = 4; 0.750 mg) 2 days prior, to determine the contribution of VEGF receptors to CNS vascular permeability. We determined that pre-treatment with DC101, but not DC6.12, significantly reduced CNS vascular permeability when compared to treatment with normal goat serum (*P* = 0.003) (Figure 1I). This further supports our hypothesis that VEGF plays a role in BBB disruption through binding receptor flk-1.

To further extend our previous study [16], we performed an analysis of VEGF receptor gene expression following induction of CD8 T cell-initiated BBB disruption*. In situ* hybridization was carried out on fresh-frozen, cryostat-cut (at 10-μm thickness), slide-mounted sections throughout the brain. Semi-adjacent sections were hybridized with ^35^S-labeled cRNA sense (control) and anti-sense probes for detection and localization of VEGF receptors flk-1 and flt-1 mRNAs according to our previously published protocol [16,17]. The flk-1 and flt-1 cDNA plasmids were contained in a pGEM3 vector and consisted of 390 bp and 660 bp, respectively (kindly provided by LF Brown, Harvard University [18]). Labeled probes were prepared by *in vitro* transcription from linearized cDNA plasmids using the proper RNA polymerase in the presence of excess ^35^S-UTP (PerkinElmer, Waltham MA, USA) and were generated as previously described [19]. The pretreated sections were incubated overnight at 60 °C in hybridization solution consisting of 50% de-ionized formamide, 10% dextran sulfate, 20 mM Tris–HCl, 1 mM EDTA, 1X Denhardt’s solution, 0.33 mg/ml denatured salmon sperm DNA, 0.15 mg/ml tRNA, 40 mM dithiothreitol, DEPC H_2_O and the ^35^S-labeled probe at a concentration of 1 × 10^6^ cpm/50 μl. After hybridization, sections were washed in a series of standard saline citrate washes including a ribonuclease A treatment. Slides were then exposed to BioMax MR film (Kodak, Rochester NY, USA) for 8 days for generation of film autoradiographs. The films were developed with Kodak GPX developer and fixer. Semi-quantitative analysis of the dorsal hippocampus (including the dentate gyrus granule cell layer, hippocampal principal cell layers and molecular layers) and striatum ipsilateral to the hemisphere of TMEV infection was performed using optical density measurements (Scion Image software, National Institutes of Health). The corrected grey levels were generated by subtracting a background measurement (taken from a non-tissue-containing area on the same slide) from the optical density measurements in the hippocampus or striatum for each section. No specific labeling was obtained with the control sense riboprobes. Mean and standard error values for *in situ* hybridization measurements were calculated using software program SigmaStat (SYSTAT Software Inc, San Jose CA, USA). Bar graphs with standard error values were plotted on software program SigmaPlot (SYSTAT Software Inc).

**Figure 2:**
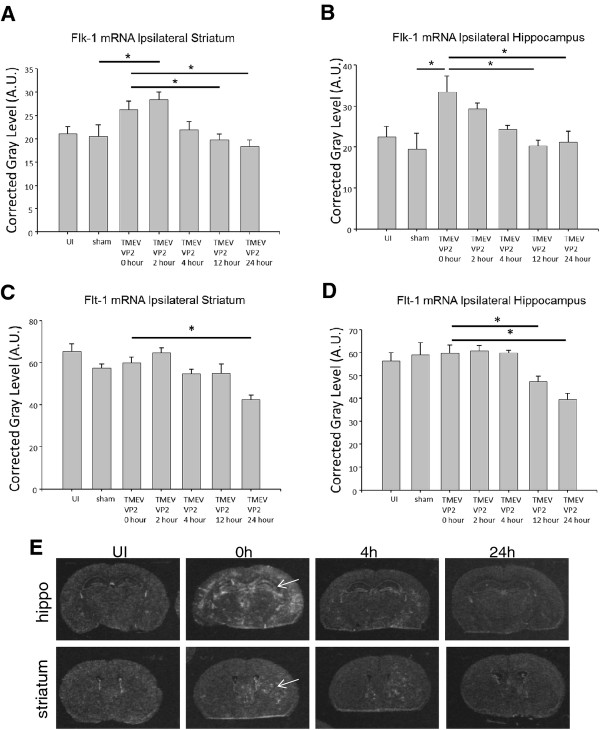
**Analysis of vascular endothelial growth factor (VEGF) receptors flk-1 and flt-1 mRNA expression in brain using *in situ *hybridization.** Flk-1 and flt-1 mRNA hybridization was determined in uninfected, sham-infected, Theiler’s murine encephalomyelitis virus (TMEV)-infected animals, and in mice at 2, 4, 12 and 24 hours post-induction of CD8 T cell-initiated BBB disruption through administration of VP2_121-130_ peptide. We present semiquantitative analysis of flk-1 expression in (**A**) the striatum and (**B**) the hippocampus of uninfected, saline-treated (sham), TMEV-infected and VP2_121-130_ peptide-administered animals. Also shown is semiquantitative analysis of flt-1 expression in (**C**) the striatum and (**D**) the hippocampus of uninfected, phosphate-buffered saline (PBS)-treated (sham), TMEV-infected and VP2_121-130_ peptide-administered animals undergoing CD8 T cell-initiated BBB disruption (*P*  < 0.05). (**E**) Representative film autoradiographs depicting flk-1 mRNA expression in the hippocampus (top row) and striatum (bottom row) of TMEV-infected mice. The expression of flk-1 mRNA appears to be altered in TMEV-injected animals prior to induction of vascular permeability (0 hours). Flk-1 mRNA levels then decline by 4 hours after induction of vascular permeability, and returned to normal levels by 24 hours (compare 24 h with UI). Arrows indicate the hippocampus (top panel) or the striatum (bottom panel). h, hours; UI, uninfected.

In Figure 2A and B, we demonstrate that at seven days post-TMEV infection, there is significantly increased expression of flk-1 mRNA in the hippocampus (n = 4 for both sham and TMEV infected animals, *P* = 0.016) and striatum (n = 4 for both sham and TMEV infected animals; *P* = 0.025) when compared to sham controls, in which sterile PBS is intracranially administered to the brain. These data also demonstrate that flk-1 mRNA expression is decreased in the hippocampus (n = 4; *P* = 0.005) and striatum (n = 4; *P* = 0.009) by 12 hours post-administration of VP2_121-130_ peptide, returning to similar expression levels as sham-treated animals. Unlike flk-1 mRNA expression, flt-1 mRNA expression in the hippocampus and striatum remains unaltered 7 days post-TMEV infection (Figure 2C and D). However, flt-1 mRNA expression is significantly decreased in the hippocampus by 12 hours post-administration of VP2_121-130_ (n = 4; *P* = 0.029; Figure 2D) and remained significantly decreased at 24 hours (n = 4; *P* = 0.002). By 24 hours, flt-1 expression is significantly decreased in the striatum (sham group n = 4, 24 hour group n = 4; *P*  < 0.001; Figure 2C). We have previously published that vascular permeability occurs as early as 4 hours post administration of VP2 peptide proportionally with the increase in VEGF cytokine expression [16]. Also in this previous study, flk-1 receptor becomes phosphorylated at 4 hours post VP2 peptide administration [16]. The reduction of flk-1 and flt-1 mRNA expression observed in this study are therefore indicative of negative regulation of VEGF receptor gene expression following increased levels of VEGF cytokine and ensuing BBB disruption in these animals.

Using this model system of immune-mediated BBB disruption, we have demonstrated that intracranial TMEV infection alters expression of flk-1, but not flt-1, mRNA in the CNS. We also show that inhibition of the VEGF co-receptor, neuropilin-1, improves survival outcome in this model. The observation that VEGF receptors contribute to vascular permeability is prominent in the literature. Studies in DHF patients have shown that vascular permeability is inversely correlated to the amount of soluble VEGFR2, the human homolog of flk-1. Levels of plasma-soluble VEGFR1, the human homolog of flt-1, were stable indicating that VEGFR2 is the key receptor involved in DHF [7]. Based on the results obtained in our model system, we hypothesize that flk-1 mRNA expression is upregulated during viral infection to promote angiogenesis and vascular permeability to enable effective inflammation necessary to clear pathogens. Furthermore, this study provides *in vivo* evidence that NRP-1, a VEGF co-receptor that has been shown to enhance flk-1 activity, is a potential target when designing therapies for neuro-inflammatory CNS conditions in which BBB integrity is compromised via VEGF-mediated mechanisms.

## Abbreviations

BBB: blood–brain barrier; AHLE: acute hemorrhagic leukoencephalomyelitis; VEGF: vascular endothelial growth factor; NRP-1: neuropilin-1; flk-1: fetal liver kinase 1; flt-1: FMS-related tyrosine kinase-1; TMEV: Theiler’s murine encephalomyelitis virus.

## Competing interests

The authors declare that they have no competing interests.

## Authors’ contributions

GLS conceived of the study, participated in design, coordination and conception of the study, drafted the manuscript, collected samples for *in situ* hybridization and optimized NRP-1 inhibitor dosing for survival, hemorrhage and permeability studies. JWD participated in design and coordination of the study and performed and analyzed the *in situ* hybridization experiments. HLJ performed volumetric image analysis of microhemorrhage and permeability. TWC executed experiments pertaining to NRP-1 inhibition and small animal MRI to detect CNS vascular permeability and microhemorrhage formation. IP participated in design and conception of the study, participated in experimental design of MRI studies. KBS participated in design and conception of the study, helped revise the manuscript for important intellectual content, and participated in the methodology of *in situ* hybridization experimentation. KDP evaluated viral loads in CNS tissue. AJJ oversaw all aspects of experimental design, execution of experiments, data analysis, and manuscript preparation. This included participating in design, coordination and conception of the study, preparation and revision of the manuscript for important intellectual content, participation in sample collection for *in situ* hybridization, and assistance with survival studies and MRI for detection of hemorrhage and permeability.
